# Case Report: Multi drug resistant tuberculosis in an 18-month-old boy with seizure, vomiting, and loss of vision

**DOI:** 10.12688/wellcomeopenres.23352.1

**Published:** 2024-11-08

**Authors:** Mukund Kumar Deo, Amod Rayamajhi, Bimala Baniya, Susan Bhattarai, Sristi Upadhyay, Sanjeet Kumar Shrestha, Buddha Basnyat, Ajit Rayamajhi

**Affiliations:** 1 Department of Pediatrics, Kanti Children's Hospital, Kathmandu, Bagmati, 44600, Nepal; 2 Global Health Research and Medical Intervention for Development, Kanti Children's Hospital, Kathmandu, Bagmati, 44600, Nepal; 3 Oxford University Clinical Research Unit Nepal, Kathmandu, Bagmati, 44600, Nepal; 4 National Academy of Medical Sciences, Kanti Children's Hospital, Kathmandu, Bagmati, 44600, Nepal

**Keywords:** Multi-drug-resistant tuberculosis, Xpert MTB/RIF Assay, Hydrocephalus, Optic atrophy, Nepal

## Abstract

Multi drug resistant tuberculosis (MDR-TB) in children is rare and requires tremendous skill and knowledge of clinicians for early detection and treatment. This case highlights the complexities in diagnosing and treating MDR-TB in young children. An 18-month-old immunocompetent boy presented with prolonged fever, sudden onset of vision loss, vomiting, and seizures, which led to a suspicion of meningitis. Given his family history of treated pulmonary TB (TB) in the father and uncle one year ago, the initial treatment followed the Nepali national protocol for non-MDR tuberculous meningitis (TBM). However, the child's lack of response to this treatment raised concerns about MDR-TB, particularly after discovering on close questioning that the uncle, who lived with the boy’s family, had actually been diagnosed with MDR-TB. The diagnosis was confirmed by molecular tests of cerebrospinal fluid (CSF) and customized treatment for MDR TBM administered. The boy then slowly improved and became less irritable, afebrile, seizure-free, developed spontaneous movements of all four limbs, and was discharged after one month. Delays in suspicion, confirmation, and treatment of MDR TBM led to complications of leptomeningitis with non-communicating hydrocephalus and bilateral optic atrophy, which was confirmed by magnetic resonance imaging (MRI) scan of the brain. This case highlights the importance of taking a very thorough and proper history and considering the possibility of MDR, especially in countries like Nepal where TB in general is rampant so that timely diagnosis and treatment is possible and complications are avoided.

## Plain language summary

Deo MK et al have managed and written up this case report entitled “Multi drug resistance tuberculosis in an 18-month-old boy with prolonged fever, seizure, vomiting, and sudden loss of vision”. The patient was admitted, diagnosed, managed and discharged from Pediatric Ward of Kanti Children’s hospital, Kathmandu, Nepal in April of 2022. If the organism, *Mycobacterium tuberculosis*, which causes tuberculosis, develops resistance against two main antitubercular drugs, rifampin and isoniazid, it can cause a disease known as multi drug resistant tuberculosis (MDR-TB). Multi drug resistant tuberculosis (MDR-TB) in children is rare and requires tremendous skill and knowledge of clinicians for early detection and treatment. Seeding of the multi drug resistance *Mycobacterium tuberculosis* in the meninges covering the brain and the spinal cord causes multi drug resistance tubercular meningitis (MDR TBM) which is diagnosed by identifying the organism in the cerebrospinal fluid (CSF) of the patient. The common clinical features are fever, headache, vomiting, loss of consciousness and seizures. The patient will not respond to the conventional treatment regime or prophylaxis tailored for non MDR TBM. Any delay in diagnosis may cause hydrocephalus and optical atrophy in the brain. We report a case of 18-month-old boy who with fever, sudden onset loss of vision, vomiting and seizures. He was suspected of the disease because of failure to respond to conventional regime and close contact with MDR TB patient. The diagnosis was confirmed by molecular tests of the CSF and customized treatment of MDR TBM administered. In countries with high prevalence of tuberculosis, MDR-TB should be suspected, investigated and treated appropriately even in children.

## Introduction

Tuberculosis (TB) is a leading cause of death worldwide. It is the most common infectious disease in the developing countries. Although it predominantly affects the lungs, extrapulmonary TB, especially central nervous system (CNS) TB, has recently increased. The three main categories of CNS TB are tuberculous meningitis (TBM), intracranial tuberculoma, and spinal tuberculous arachnoiditis ^1^. TBM accounts for approximately 1% of all TB cases and 5% of all extrapulmonary cases in immunocompetent individuals, often leading to severe complications ^2^.

If *Mycobacterium tuberculosis* becomes resistant to major antitubercular drugs such as rifampin and isoniazid, it can cause a disease known as multidrug-resistant tuberculosis (MDR-TB). MDR-TB is not only a threat to global health security but also the Achilles heel of the tuberculosis control program of most developing countries, posing significant challenges. Patients with MDR-TB are difficult to treat because their diagnostic capabilities in Nepal are limited, medicines are limited, are often unavailable, and are unaffordable. The most common reasons for MDR-TB to emerge and spread are mismanagement of the “garden variety” of TB patients and overcrowded living conditions ^3^.

In children, MDR-TB is rare and requires special clinical skills and knowledge from clinicians for early detection and effective management. A frequent complication of TBM is hydrocephalus, which can be either communicating or non-communicating. Hydrocephalus can cause seizures or visual loss owing to optic atrophy ^4, 5^.

## Case report

An 18-month-old child presented with a history of fever for four weeks along with abnormal body movements, vomiting, and sudden onset of vision impairment in both eyes for five days. The child was irritable and could not recognize parents or other objects. There was no cough, weight loss, ear discharge, facial deviation, or nasal regurgitation. A family history of pulmonary TB in the father and uncle one year ago, both appropriately treated, importantly (as more focused history revealed later) the uncle was found to have MDR-TB and treated accordingly, during which time the child had received oral isoniazid (at 15 mg/kg/day) once daily for 6 months as prophylaxis against pulmonary TB.

On examination, he was pale, irritable, with a pulse of 130 beats/minute, respiratory rate of 18/minute, blood pressure (BP) of 70/40 mmHg in the right arm in the supine position, and capillary refill time of 2 seconds. However, the child had normal anthropometry (weight, 10 kg; length, 86 cm) and delayed fine and gross motor skills, because he walked with support. There was neck rigidity, positive Babinski reflex, bilaterally reactive pupils, increased tone, and up-going plantar reflexes in both the lower limbs. Ocular examination revealed a bilateral pale disc with well-demarcated margins and vessels, suggestive of bilateral optic atrophy. Extraocular movements of the eyes, cranial nerves, and sensorimotor system were normal.

**Figure 1.**
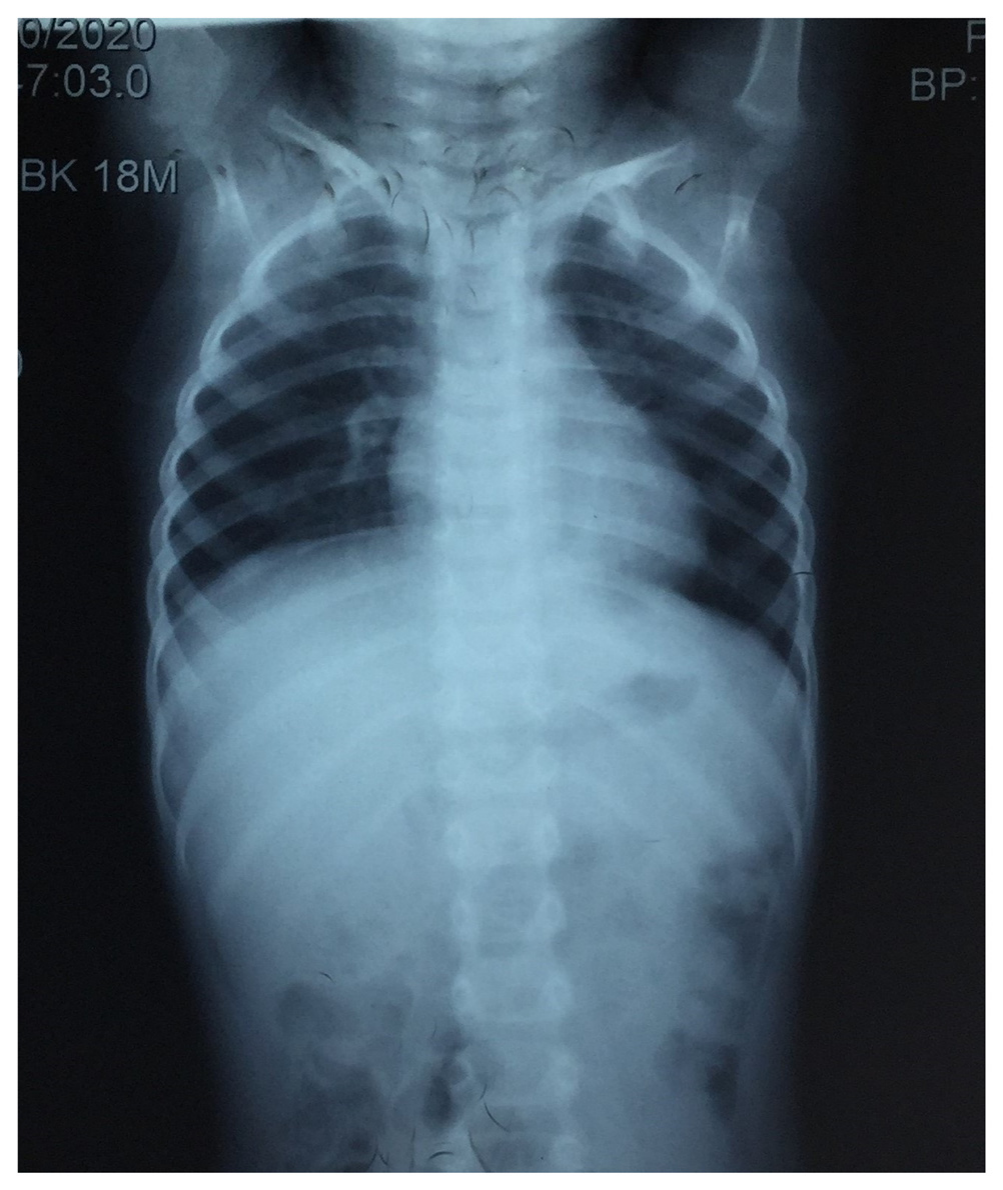
Chest radiograph showing normal bilateral lung fields, cardiophrenic and costophrenic angles, and cardiac shadow.

**Figure 2.**
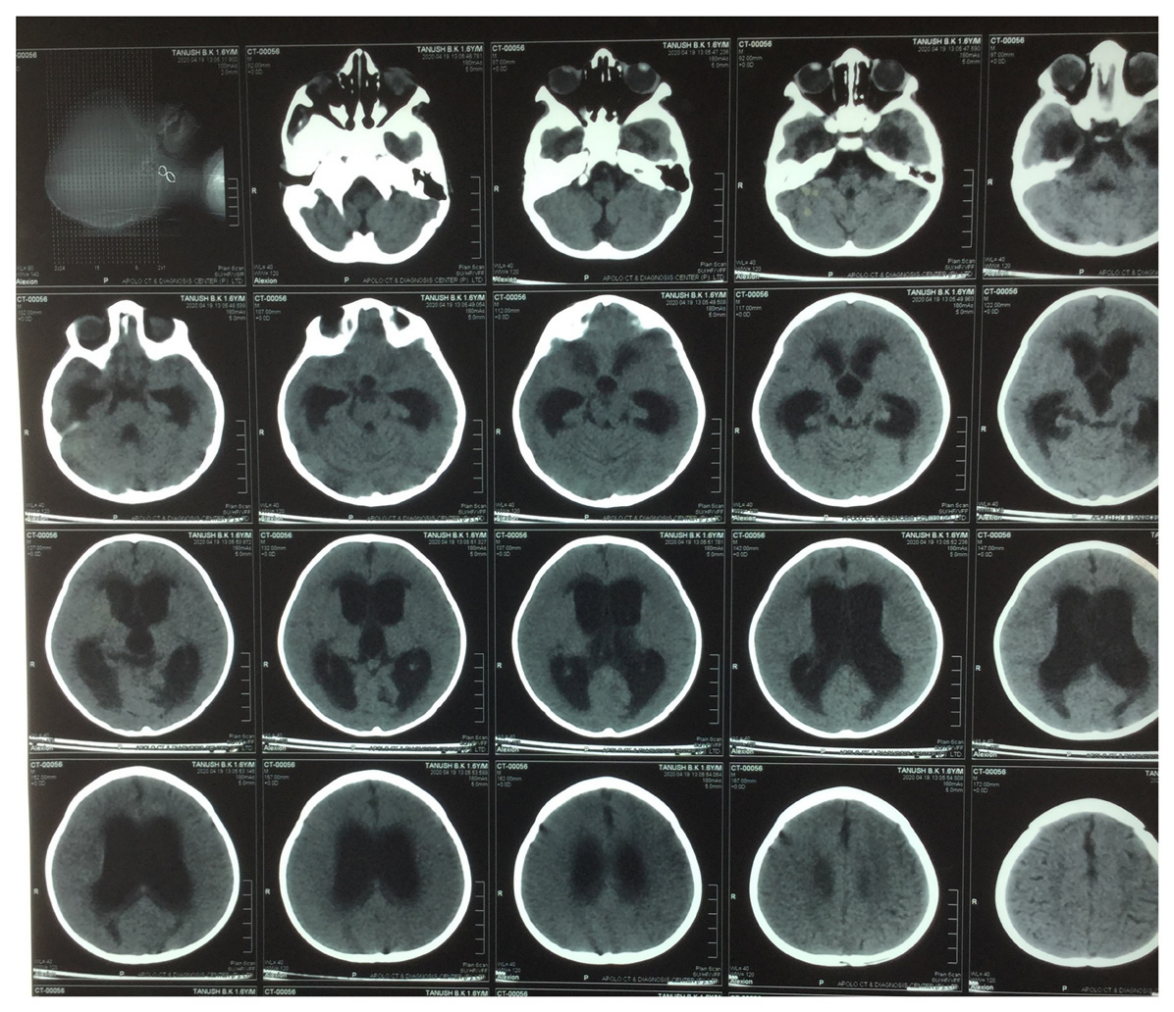
Plain computed tomography (CT) of the brain showed moderately dilated bilateral lateral and third ventricles with non-dilated fourth ventricle and periventricular oozing in the bilateral frontal, temporal, and occipital horn, suggestive of non-communicating hydrocephalus.

**Figure 3.**
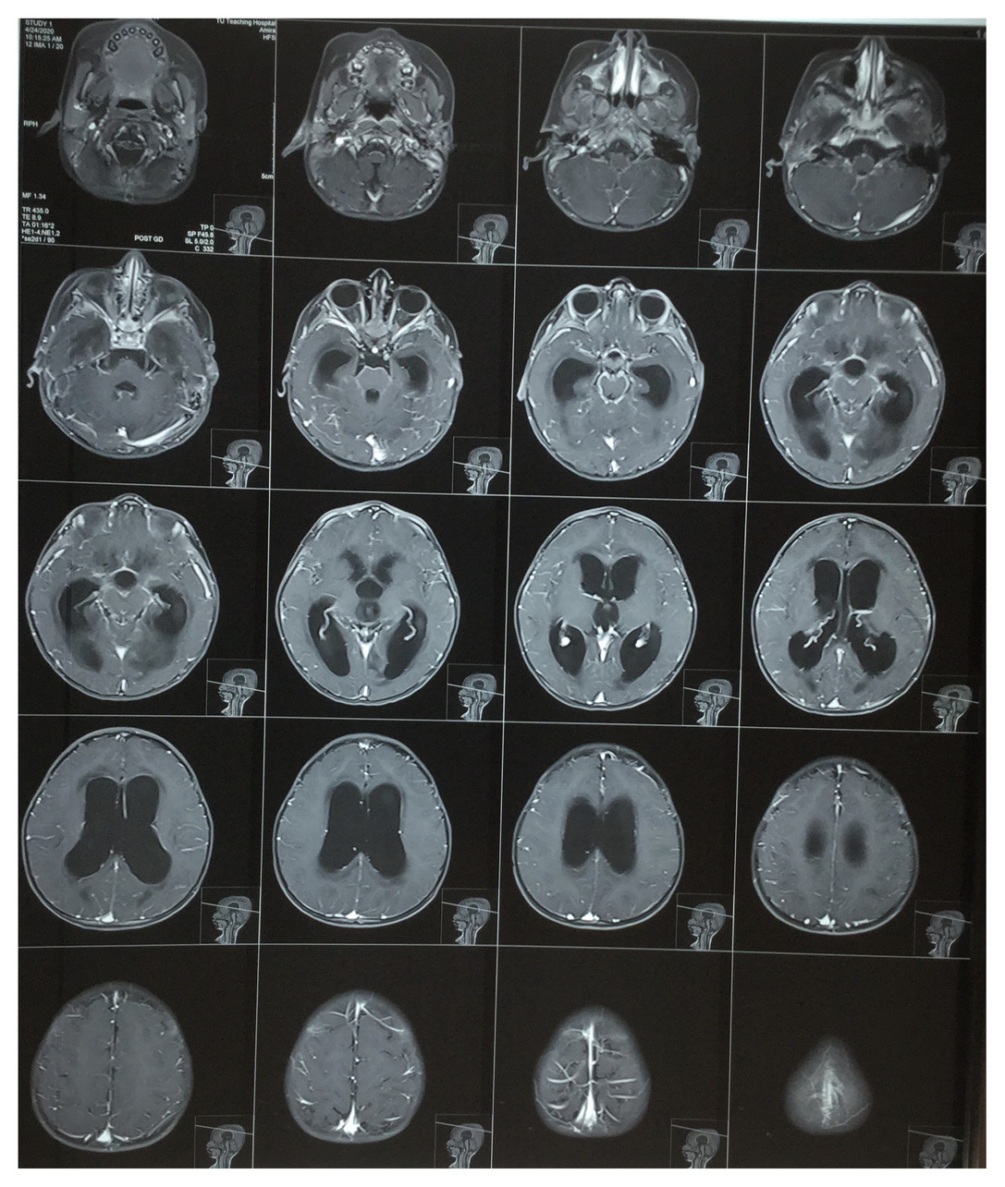
Contrast-enhanced magnetic resonance imaging (MRI) of the brain also showed features of noncommunicating hydrocephalus. Leptomeningeal enhancement was seen in the bilateral temporal regions, suggestive of leptomeningitis with aqueduct obstruction with exudates due to meningitis.

Hematological investigations revealed microcytic hypochromic anemia (Hemoglobin 9.8 gm/dl) and an elevated erythrocyte sedimentation rate of 52 mm/h. Renal and liver function test results were within normal limits. There was no growth of any organism in blood or urine bacterial cultures. Chest radiography revealed normal findings ( Figure 1). Tuberculin skin test results were normal. Cerebrospinal fluid (CSF) analysis revealed a normal appearance, and the total leukocyte count (TLC) was 260 cells/mm ^3^ (neutrophils 40% and lymphocytes 60%), protein 118 mg/dl, and sugar 42 mg/dl. No organism was observed on Gram staining, acid-fast bacilli (AFB) staining, or bacterial culture of CSF. The CSF Adenosine deaminase (ADA) was 30 U/L. Gene Xpert testing was unavailable at this time. Gastric lavage for *Mycobacterium tuberculosis* did not yield any organism. Plain Computed Tomography (CT) of the brain revealed non-communicating hydrocephalus ( Figure 2). Contrast-enhanced magnetic resonance imaging (MRI) of the brain showed features suggestive of leptomeningitis with non-communicating hydrocephalus and aqueduct obstruction with exudates due to meningitis ( Figure 3).

Irritability and cognitive disorientation, along with a family history of pulmonary TB in the father and uncle one year ago and an available investigation report raised significant suspicion for a central nervous system infection, potentially tuberculous meningitis (TBM). Hence, the child was started on anti-tubercular treatment (ATT) with oral isoniazid (10 mg/kg/day) once daily, oral rifampicin (15 mg/kg/day) once daily, oral pyrazinamide (25 mg/kg/day) once daily, and intramuscular injection of streptomycin (20 mg/kg/day). Ethambutol was not used because of fear of further deterioration of vision due to optic neuritis. Intravenous dexamethasone (0.6 mg/kg/day) was administered for 6 h and oral acetazolamide (25 mg/kg/day) for 8 h for raised intracranial pressure (ICP) and intravenous sodium valproate (20 mg/kg/day) for 12 h for seizure. ATT was planned for 12 months according to the National TB guideline of Nepal ^6^. A ventriculoperitoneal shunt placement was also planned by the neurosurgeons to improve and stabilize the clinical condition. Other complications were monitored and supportive care was provided as necessary.

Since the clinical condition of the patient did not show any improvement after nine days, a closer re taking of the history revealed that the uncle had MDR-TB. A repeat guarded lumbar puncture (LP) was performed on the tenth day after starting ATT. This time, the Xpert MTB/RIF assay was available for CSF analysis. The CSF cytology and biochemistry results were similar to those of previous CSF findings. However, the CSF Xpert MTB/RIF assay reported *Mycobacterium tuberculosis* with rifampicin resistance. Since 95% of patients with rifampicin resistance have concurrent resistance to isoniazid, we considered rifampicin resistance to be a surrogate marker of MDR-TB ^7^. The diagnosis was then revised to MDR-TB and ATT stopped. The modified MDR-TB regimen consisted of linezolid (10 mg/kg/day), levofloxacin (20 mg/kg/day), clofazimine (3 mg/kg/day), and pyrazinamide (30 mg/kg/day). Intravenous dexamethasone (0.6 mg/kg/day) 6 h and oral pyridoxine (10 mg) once a day were also included ^6^. Over the following weeks, the patient exhibited notable clinical improvement. His sensorium improved. Vital signs such as respiration, pulse rate, BP, and capillary perfusion returned to normal. The patient became less irritable, afebrile, and seizure-free. There was also an improvement in tone, power, reflexes, and spontaneous movements of all four limbs. A repeat CSF analysis three weeks later demonstrated a significant reduction in total TLC to 50/ mm ^3^ (neutrophils 2%, lymphocytes 98%), sugar 54 mg/dl, protein 64 mg/dl, and ADA 11.1 U/L, indicating a positive response to therapy. Hence, the patient stabilized with the ATT regimen, which was planned for a total duration of 18 months. The patient was discharged with instructions for regular follow-up every three months to assess efficacy and monitor complications.

## Discussion

Worldwide, it is estimated that approximately 10 million people suffer from tuberculosis every year with 1.1 million cases in children. Two-thirds of the total new cases were concentrated in eight countries, primarily located in South Asia. In Nepal, between 350 and 450 cases of MDR-TB are reported every year ^7^. Diagnosing MDR-TB in children requires a high index of suspicion and can be particularly challenging.

It was unfortunate that Gene Xpert testing was unavailable the first time LP was done, which would have given us the correct diagnosis earlier. If a proper and detailed history had been taken earlier, treatment for MDR-TB could have been instituted earlier, which may have prevented the patient from developing hydrocephalus and bilateral optic atrophy. 

Hydrocephalus is a common complication of TBM, affecting approximately two-thirds of the patients. This condition often results from a dysregulated immune response to infection, leading to obstruction of CSF pathways ^8^. It has been reported to be an associated feature in 65% of TBM patients at the time of initial presentation to the health facilities, emphasizing the need for early intervention ^9^. Factors which increase the risk of hydrocephalus include advanced stage of TBM, duration of illness of more than 2 months, CSF total cell count more than 100/mm ^3^ and CSF protein more than 2.5 g/L ^9^. Addressing these issues through effective screening, early diagnosis, and appropriate treatment, is essential to improve outcomes.

Optic atrophy is reported in 27–72 % patients with TBM ^10^. It can cause irreversible loss of eye sight. In TBM, it can result from various pathological processes, such as compression of the optic chiasm due to hydrocephalus, inflammation and ischemia of the optic nerves because of arachnoiditis, papilledema from increased intracranial pressure, occipital infarction due to vasculitis, and toxic effects of anti-tuberculosis medication ^11^. Hence, the associated visual defect depends not only on the site and extent of the disease in the CNS, but also on the therapeutic agent being administered to a child with TBM.

## Conclusion

Diagnosing MDR-TB in children is challenging and requires a high level of suspicion. It is essential to identify *Mycobacterium tuberculosis* in the CSF through bacterial culture, as well as using rapid diagnostic methods such as the Xpert MTB/RIF assay, for the diagnosis of MDR TBM.

Patients with MDR TBM may present to health facilities with symptoms and signs of obstructive hydrocephalus, which may require shunt placement, in addition to appropriate ATT. Additionally, optic atrophy resulting from TBM can cause sudden onset of visual impairment. However, the most important take-home message for us was to not forget to focus on taking a detailed history against the background of this rampant disease in Nepal, especially in the context of unusual presentation of this pediatric patient with severe meningitic features, so that MDR-TB is not missed and treated effectively.

## Consent

Both verbal and written informed consent were obtained from the parents for publication of this clinical details and clinical images.

## Data Availability

No data are associated with this article
