# Supplementary material for: Case Report: Multi drug resistant tuberculosis in an 18-month-old boy with seizure, vomiting, and loss of vision
Source: Wellcome Open Res. 2025 Oct 15;9:669. Originally published 2024 Nov 8. [Version 2] doi: 10.12688/wellcomeopenres.23352.2 (PMC12743214; doi:10.12688/wellcomeopenres.23352.2)
Supplement: Peer review history

# Reviewer report: Reviewer response for version 2

**DOI:** 10.21956/wellcomeopenres.27678.r136367

**Published:** 2026-03-06

**Authors:** Sachin Atre

**Affiliations:** 1 Dr. D.Y. Patil Medical College, Hospital and Research Centre, Pune, India

Authors have responded to all my queries. Only one correction needs to be done. 6 months of daily levofloxacin is recommended as prophylaxis for contacts of MDR-TB cases, if index case is not fluroquinolone resistant. If INH monoresisatnce is there, then 4 months of rifampicin is recommended.

Are enough details provided of any physical examination and diagnostic tests, treatment given and outcomes?

Yes

Is the case presented with sufficient detail to be useful for other practitioners?

Yes

Is sufficient discussion included of the importance of the findings and their relevance to future understanding of disease processes, diagnosis or treatment?

Partly

Is the background of the case’s history and progression described in sufficient detail?

Partly

Reviewer Expertise:

TB and MDR-TB

I confirm that I have read this submission and believe that I have an appropriate level of expertise to confirm that it is of an acceptable scientific standard.

# Reviewer report: Reviewer response for version 2

**DOI:** 10.21956/wellcomeopenres.27678.r143080

**Published:** 2026-01-10

**Authors:** Dejair Caitano do Nascimento

**Affiliations:** 1 Basic Research Center, Porto Alegre, Brazil

**Manuscript Report**

 Title: In my opinion, the title should show one of the important aspects of the manuscript, which is "MDR TBM led to complications of leptomeningitis with hydrocephalus and bilateral optic atrophy," or highlight the CSF GeneXpert testing to confirm the early diagnosis and start the treatment.

**Abstract:** The abstract is well-written and clearly describes the main important points of the manuscript.

**Case report**: The case report describes the case in detail, considering the complete information about the patient's clinical history; vital signs, laboratory test data, imaging exams (chest radiograph, CT and MRI of the brain), and CSF GeneXpert, which was most important to confirm the diagnosis.

**Conclusion: **Considering that it was the CSF GeneXpert test that confirmed the diagnosis, this should be discussed further in the manuscript, as well as epidemiological aspects of TB in Nepal.

 Here is a suggestion for the authors. However, the manuscript is important for training professionals working in public health in a country endemic for TB.

Are enough details provided of any physical examination and diagnostic tests, treatment given and outcomes?

Yes

Is the case presented with sufficient detail to be useful for other practitioners?

Yes

Is sufficient discussion included of the importance of the findings and their relevance to future understanding of disease processes, diagnosis or treatment?

Yes

Is the background of the case’s history and progression described in sufficient detail?

Yes

Reviewer Expertise:

I am a scientific researcher. My area of ​​expertise is pharmacology. Specifically drugs used in the treatment of infectious dermatological diseases and In particular, drugs used in the treatment of leprosy.

I confirm that I have read this submission and believe that I have an appropriate level of expertise to confirm that it is of an acceptable scientific standard.

# Reviewer report: Reviewer response for version 2

**DOI:** 10.21956/wellcomeopenres.27678.r138335

**Published:** 2025-12-26

**Authors:** Milagros Zavaleta

**Affiliations:** 1 Research and development, BTS Consultores S.A.C., Lima , 15082, Lima, Peru

**Your Report**

 Please provide a full report, expanding on your answers to the questions above. In particular, if you answered “no” or “partly” to any of the questions, please give constructive and specific details as to how the authors can address any criticisms. Please indicate clearly which points must be addressed to make the article scientifically sound. (50 words min.)

**Introduction section**

 The authors state that “In children, MDR-TB is rare,” and the aim of the case report is to “highlight the complexities in diagnosing and treating MDR-TB in young children.” Given this objective, it is important that the authors quantify the proportion of children affected by TB and MDR-TB in Nepal. Including these epidemiological data would strengthen the introduction and clarify the relevance of this case within the national context.

**Case Report Section**

  

- Please indicate the time required for the hospital to perform the brain CT and MRI, including the time from the child’s arrival to acquisition, analysis, and reporting of the results. This information is important for evaluating delays in early diagnosis, particularly considering that GeneXpert testing was not available at first presentation.

 

- Reference 6 does not correctly link to the statement: “Since 95% of patients with rifampicin resistance have concurrent resistance to isoniazid, we considered rifampicin resistance to be a surrogate marker of MDR-TB.” Please verify and cite the correct source.

**Discussion Section**

  

- Include a discussion on the relevance of thorough anamnesis. Given the history of close contact with an MDR-TB case, as well as the child’s prior preventive therapy, more rigorous history-taking at the time of admission could have guided earlier suspicion of MDR-TB. If the child had previously been treated at the same health facility, the clinical history should have been reviewed more closely.

 

- There appears to be a social component affecting the quality of care provided to the patient. This lack of attention in patient tracing, combined with limited diagnostic capacity, contributed to deterioration of the child’s clinical condition. Expanding on this dimension would strengthen the discussion.

 

- Although the authors mention the unavailability of GeneXpert, it would be valuable to comment on the need for new, cost-effective molecular resistance-detection methods—particularly in low-resource settings such as Nepal where high-cost systems like GeneXpert (and their consumables) are difficult to sustain. These limitations delay early and accurate diagnosis.
- While GeneXpert detects rifampicin resistance and correlates with concurrent INH resistance in many cases, more specific resistance-detection methods remain necessary to guide appropriate treatment—especially given the toxicity of MDR-TB medications. The article does not explain limitations of Gen Xpert nor the need for DST/line-probe assays for accurate management.

 

- If the child had received isoniazid previously, the case should also highlight failures in anamnesis rather than focusing solely on GeneXpert unavailability. Although diagnostic limitations are an important systemic issue in developing countries, this case illustrates that detailed clinical history—including previous treatment and MDR-TB exposure—is equally essential for early diagnosis and proper management. The authors should explore this aspect in greater depth.

 

- Please also discuss the treatment regimen described in the case report:
  “oral isoniazid (10 mg/kg/day), rifampicin (15 mg/kg/day), pyrazinamide (25 mg/kg/day), and intramuscular streptomycin (20 mg/kg/day); ethambutol was withheld due to concern for optic neuritis.” Explain its relevance to drug-resistance detection, especially given the authors’ concern regarding potential adverse effects in a child with vision loss.

**Conclusion Section**

  

- Consider reinforcing the need for MDR-TB diagnostic methods that are affordable in low-resource health systems such as Nepal. Improving diagnostic access would enhance early detection and treatment, consistent with the authors’ own observations regarding resource limitations.

 

- The authors appropriately emphasize the importance of taking a detailed history; however, the analysis does not clarify whether the failure in this case was due to insufficient depth of clinical assessment or the need for a more systematic and validated approach to history-taking. This should be further elaborated.

**“**I have read this submission. I believe that I have an appropriate level of expertise to confirm that it is of an acceptable scientific standard, however, I have a number of small changes to the article, or specific, sometimes more significant revisions.”

Are enough details provided of any physical examination and diagnostic tests, treatment given and outcomes?

Yes

Is the case presented with sufficient detail to be useful for other practitioners?

Yes

Is sufficient discussion included of the importance of the findings and their relevance to future understanding of disease processes, diagnosis or treatment?

Partly

Is the background of the case’s history and progression described in sufficient detail?

Yes

Reviewer Expertise:

Molecular diagnostics test for infectious diseases (TB, leptospirosis, malaria, COVID-19) and neglected diseases in low-income countries.

I confirm that I have read this submission and believe that I have an appropriate level of expertise to confirm that it is of an acceptable scientific standard, however I have significant reservations, as outlined above.

# Reviewer report: Reviewer response for version 2

**DOI:** 10.21956/wellcomeopenres.27678.r136368

**Published:** 2025-10-25

**Authors:** Hussein H Twabi

**Affiliations:** 1 Kamuzu University of Health Sciences, Blantyre, Malawi

No further comments

Are enough details provided of any physical examination and diagnostic tests, treatment given and outcomes?

Partly

Is the case presented with sufficient detail to be useful for other practitioners?

Partly

Is sufficient discussion included of the importance of the findings and their relevance to future understanding of disease processes, diagnosis or treatment?

No

Is the background of the case’s history and progression described in sufficient detail?

Partly

Reviewer Expertise:

TB/HIV epidemiology, TB drug trials, diagnostic trials, Paediatric TB, digital health solutions for infectious diseases, and Infectious disease epidemiology in low resource settings.

I confirm that I have read this submission and believe that I have an appropriate level of expertise to confirm that it is of an acceptable scientific standard.

# Reviewer report: Reviewer response for version 1

**DOI:** 10.21956/wellcomeopenres.25739.r115031

**Published:** 2024-12-13

**Authors:** Hussein H Twabi

**Affiliations:** 1 Kamuzu University of Health Sciences, Blantyre, Malawi

Background section:

 The authors need to revise the opening statement of the introduction "tuberculosis is a leading cause of death worldwide" to clarify that TB is the leading infectious cause of death. Additionally, the 2nd sentence is false: infections such as malaria, URTIs and rotavirus far exceed TB. Otherwise the authors need to provide citations for these claims. The paper citing the last sentence of paragraph 1 (citation 2) does not contain the statistics that have been reported. As such, the authors need to review their citations and ensure that they are real and traceable. The authors need to ensure that the arguments presented in the background follow a reasonable and coherent flow (this is especially evident for paragraph 2 and 3 of the introduction). The last paragraph of the introduction requires a conclusive rationale sentence to tie it together.

 Case report section:

 The audience for this article is largely technocratic. As such, the authors may as well use technical terms that best describe the symptoms. This is in reference to the "abnormal body movements" in paragraph 1 under the subheading case report. What are these abnormal body movements as there can be a myriad of them which describe different disease processes, such as chorea, tonic clonic movements, myoclonic jerks, intention tremors, and so forth.

 The authors need to rewrite the last sentence of the first paragraph under case report as it contains a sentence fragment. 

 The paragraph talking about examination findings do not report any standardised neurological exam that elucidates cognitive disorientation in 18 month old babies. As such, how was this determined as reported in the 4th paragraph under case report subheading?

 In paragraph 5 under the case report subheading, the authors state that "95% of patients with rifampicin resistance have concurrent resistance to isoniazid" and cited a paper from Ethiopia to back this claim. This paper does not report the prevalence of isoniazid resistance amongst Rif-resistant cases. The authors thus need to review this citation and cite the correct paper. Additionally, was a repeat Xpert MTB/RIF of the CSF conducted at week 4 (3 weeks later) as part of TB treatment response monitoring?

 Discussion section:

 Paragraph 1 under the discussion subheading cites the Ethiopia paper in defence of a claim that there are between 350 and 450 MDR-TB cases each year. The paper is from Ethiopia and does not report the burden of MDR-TB in Nepal. The authors need to cite the correct paper here (such as the WHO Global TB Report 2024). Additionally, this paragraph does not present coherent discussion points and jumps from burden of disease to stating that diagnosing MDR-TB in children is difficult. The authors should rewrite it to provide a better flow of arguments.

 Paragraph 2 of the discussion does not present any discussion points but rather describes the limitation of the case management as was experienced by the authors. It should either be rewritten as a discussion or removed from this section. 

 Paragraph 3 and 4 of the discussion present hydrocephalus and optic atrophy as one would in the introduction. The authors need to rewrite these as discussion paragraphs.

Are enough details provided of any physical examination and diagnostic tests, treatment given and outcomes?

Partly

Is the case presented with sufficient detail to be useful for other practitioners?

Partly

Is sufficient discussion included of the importance of the findings and their relevance to future understanding of disease processes, diagnosis or treatment?

No

Is the background of the case’s history and progression described in sufficient detail?

Partly

Reviewer Expertise:

TB/HIV epidemiology, TB drug trials, diagnostic trials, Paediatric TB, digital health solutions for infectious diseases, and Infectious disease epidemiology in low resource settings.

I confirm that I have read this submission and believe that I have an appropriate level of expertise to state that I do not consider it to be of an acceptable scientific standard, for reasons outlined above.

# Response

**Published:** 2025-10-06

**Authors:** Ajit Rayamajhi

**Affiliations:** Pediatrics, Kanti Children's Hospital, Kathmandu, Bagmati, Nepal

1.The authors need to revise the opening statement of the introduction "tuberculosis is a leading cause of death worldwide" to clarify that TB is the leading infectious cause of death. Answer: Thank you. We have changed the opening statement in introduction as - Tuberculosis is one of the leading infectious causes of death worldwide.

 2. Additionally, the 2nd sentence is false: infections such as malaria, URTIs and rotavirus far exceed TB. Otherwise the authors need to provide citations for these claims Answer: Thank you. In first paragraph, we have changed the second line in introduction as- It is one of the most significant infectious diseases in the developing countries.

 3. The paper citing the last sentence of paragraph 1 (citation 2) does not contain the statistics that have been reported. As such, the authors need to review their citations and ensure that they are real and traceable. Answer: Thank you for pointing out. The last sentence of paragraph 1 is- TBM accounts for approximately 1% of all TB cases and 5% of all extrapulmonary cases in immunocompetent individuals, often leading to severe complications2. Hence the reference 2 has been changed to- Audrey Wanger, Violeta Chavez, Richard S.P. Huang, Amer Wahed, Jeffrey K. Actor, Amitava Dasgupta, Chapter 3 - Specific Clinical Infections, Editor(s): Audrey Wanger, Violeta Chavez, Richard S.P. Huang, Amer Wahed, Jeffrey K. Actor, Amitava Dasgupta, Microbiology and Molecular Diagnosis in Pathology, Elsevier, 2017, Pages 21-50, https://doi.org/10.1016/B978-0-12-805351-5.00003-X.

 4. The authors need to ensure that the arguments presented in the background follow a reasonable and coherent flow (this is especially evident for paragraph 2 and 3 of the introduction). The last paragraph of the introduction requires a conclusive rationale sentence to tie it together. Answer: Thank you for the comment. Hence, we have added last sentence in last paragraph of introduction section as – Hence, MDR-TB needs to be suspected, investigated and treated, even in children, in order to avoid such devastating consequences.   5. The audience for this article is largely technocratic. As such, the authors may as well use technical terms that best describe the symptoms. This is in reference to the "abnormal body movements" in paragraph 1 under the subheading case report. What are these abnormal body movements as there can be a myriad of them which describe different disease processes, such as chorea, tonic clonic movements, myoclonic jerks, intention tremors, and so forth. Answer: Thank you. We thought we would write in the patient’s own language because it was presenting complaint. However, we replaced the word abnormal body movement with “generalized tonic clonic seizure" in the first paragraph of case report section.

 6. The authors need to rewrite the last sentence of the first paragraph under case report as it contains a sentence fragment. Answer: Thank you. We have re-written the last sentence of first paragraph of case report as- There was family history of pulmonary TB in the father one year ago, which was appropriately treated. Importantly, more focused history at a later date revealed that his uncle had MDR-TB one year ago and was also treated accordingly. During which time the child had also received oral isoniazid (at 15 mg/kg/day), once daily, for 6 months as preventive therapy against pulmonary TB.

 7. The paragraph talking about examination findings do not report any standardized neurological exam that elucidates cognitive disorientation in 18-month-old babies. As such, how was this determined as reported in the 4th paragraph under case report subheading? Answer: Thank you. On second paragraph of case report from fifth line we have clarified standardized neurological examination conducted to asses cognition as- On neurological examination based on Denver Development Screening Test -II, he appeared irritable, showed poor interaction with caregiver and did not follow simple commands. He was also unable to name or point at different body parts and wore a blunted facial expression. He had neck rigidity and positive Brudzinski's reflex. Tone was increased in all four limbs with upgoing both plantar reflexes. Pupils were bilaterally normal in size but were sluggish in reaction. Fundoscopy revealed bilateral pale disc with well demarcated margins and vessels, suggestive of bilateral optic atrophy. Rest of the neurological and systemic examinations were normal.

 8. In paragraph 5 under the case report subheading, the authors state that "95% of patients with rifampicin resistance have concurrent resistance to isoniazid" and cited a paper from Ethiopia to back this claim. This paper does not report the prevalence of isoniazid resistance amongst Rif resistant cases. The authors thus need to review this citation and cite the correct paper. Answer: Thank you. We apologize for wrong citation. It should be reference 6 rather than 7. We have corrected the citation as- Since 95% of patients with rifampicin resistance have concurrent resistance to isoniazid, we considered rifampicin resistance to be a surrogate marker of MDR-TB6.

 9. Additionally, was a repeat Xpert MTB/RIF of the CSF conducted at week 4 (3 weeks later) as part of TB treatment response monitoring? Answer: Thank you for the question. Although there was no clear guideline on when to repeat CSF in MDR-TB, it was done 3 weeks later, as a part of TB treatment response monitoring. Hence, we have made correction to the third last line of the last paragraph of case report section as- A repeat CSF analysis three weeks later, conducted for treatment response monitoring, demonstrated a significant reduction in total TLC to 50/ mm 3 (neutrophils 2%, lymphocytes 98%), sugar 54 mg/dl, protein 64 mg/dl, and ADA 11.1 U/L, indicating a positive response to therapy.

 10. Paragraph 1 under the discussion subheading cites the Ethiopia paper in defence of a claim that there are between 350 and 450 MDR-TB cases each year. The paper is from Ethiopia and does not report the burden of MDR-TB in Nepal. The authors need to cite the correct paper here (such as the WHO Global TB Report 2024). Answer: Thank you for the suggestion. We have added reference 6 as citation. The reference 6 is- National guideline on drug resistant TB management. In: Services DoH, editor. Thimi, Bhaktapur, Nepal: Ministry of Health and Population. 2019; 1–186. Hence, we have added reference 6 as- In Nepal, 350 to 450 cases of multidrug-resistant tuberculosis (MDR-TB) are reported each year, and in 2023 the World Health Organization classified Nepal among the top 30 countries with the highest multidrug resistant tuberculosis (MDR/RR-TB) burden 6.7.

 11. Additionally, this paragraph does not present coherent discussion points and jumps from burden of disease to stating that diagnosing MDR-TB in children is difficult. The authors should rewrite it to provide a better flow of arguments. Answer: Thank you. We have rewritten the first paragraph in Page 6 as- Tuberculosis continues to be a major global health problem, affecting an estimated 10 million people annually, including 1.3 million children (https://www.who.int/news-room/fact-sheets/detail/tuberculosis). Nearly two-thirds of all new cases are concentrated in just eight countries, most of which are in South Asia. In Nepal, 350 to 450 cases of multidrug-resistant tuberculosis (MDR-TB) are reported each year, and in 2023 the World Health Organization classified Nepal among the top 30 countries with the highest multidrug resistant tuberculosis (MDR/RR-TB) burden 6.7. While the treatment success rate for drug-susceptible TB remains high, outcomes for MDR/RR-TB are far less encouraging, with only 71% of cases achieving successful treatment.

 12. Paragraph 2 of the discussion does not present any discussion points but rather describes the limitation of the case management as was experienced by the authors. It should either be rewritten as a discussion or removed from this section. Answer: Thank you. We have rewritten paragraph 2 of discussion as- Diagnosis of MDR-TB in children, remains a major challenge. In this case, earlier recognition of MDR-TB meningitis might have been possible with a more detailed exposure history and initial CSF GeneXpert testing. The test was temporarily unavailable due to a shortage of cartridge in the hospital supply chain, which became accessible only after the supply chain improved. Sometimes delay in repair of the broken machine and lack of trained human resources, also causes unavailability of the test. The father’s history of drug-susceptible TB and prior isoniazid preventive therapy created a false sense of protection, delaying suspicion of resistant disease. Subsequent history of exposure to an uncle with MDR-TB ultimately clarified the diagnosis, emphasizing the importance of detailed history taking and meticulous contact tracing. This case also highlights the ineffectiveness of isoniazid prophylaxis against MDR-TB; instead, 6 months of daily levofloxacin and 4 months of daily rifampicin is recommended for such contacts. Finally, the limited availability of GeneXpert testing and trained personnel in resource-constrained settings continues to impede timely diagnosis and treatment.

 13. Paragraph 3 and 4 of the discussion present hydrocephalus and optic atrophy as one would in the introduction. The authors need to rewrite these as discussion paragraphs. Answer: Thank you for the suggestion. We have rewritten the third paragraph of discussion section as- Hydrocephalus is a well-recognized complication of TBM, reported in up to two-thirds of affected patients. It is thought to arise from a dysregulated host immune response to infection, leading to obstruction of CSF pathways8. Previous studies note that nearly 65% of patients present with hydrocephalus at initial evaluation, emphasizing the importance of timely recognition and management9. Risk factors for hydrocephalus include advanced stage of TBM, prolonged illness exceeding two months, elevated CSF total cell counts (>100/mm³), and high CSF protein levels (>2.5 g/L)9. Early diagnosis, effective screening, and prompt initiation of appropriate therapy are therefore crucial to improve outcomes in this vulnerable group.   We have also rewritten the fourth paragraph of the discussion section as- Optic atrophy is a frequent complication of TBM, reported in 27–72% of patients, and often leads to irreversible visual loss10. The underlying mechanisms are diverse, including optic chiasmal compression from hydrocephalus, arachnoiditis-related inflammation and ischemia, papilledema from raised intracranial pressure, occipital infarction due to vasculitis, and drug-related toxicity11. Hence, the severity and pattern of visual impairment reflect both the site and extent of CNS involvement as well as the effects of anti-tuberculosis therapy. message is unclear or incomplete. Please provide more details or context.

# Reviewer report: Reviewer response for version 1

**DOI:** 10.21956/wellcomeopenres.25739.r113191

**Published:** 2024-12-06

**Authors:** Sachin Atre

**Affiliations:** 1 Dr. D.Y. Patil Medical College, Hospital and Research Centre, Pune, India

1. The case study highlights the importance of history taking and early suspicion of MDR-TB which can help diagnose and treat the children with TBM or other types of TB.
2. It will be useful if authors can provide details of global burden of TB and MDR-TB among children in the introduction rather than in as the first para in the discussion section.
3. Instead of using “major anti-TB drugs”, authors can use “most potent first-line anti-TB drugs”
4. MDR-TB is not just health system or drug related issue, but the drugs used for treating MDR-TB are toxic, with long-term and permanent side-effects such as loss of hearing etc.
5. The case study also brings one of the important issues that the INH prophylaxis in this case did not offer protection against TB or MDR-TB. Also, the contacts of MDR-TB are not offered INH prophylaxis. It should be 6 months of daily levofloxacin and 4 months of daily rifampicin
6. WHO estimates that there are 1.25 million children with TB. This figure needs to be updated with the reference to the WHO website.
7. Discussion can add details about why Xpert was not available initially and how they suddenly become available. These are systems issue.

Are enough details provided of any physical examination and diagnostic tests, treatment given and outcomes?

Yes

Is the case presented with sufficient detail to be useful for other practitioners?

Yes

Is sufficient discussion included of the importance of the findings and their relevance to future understanding of disease processes, diagnosis or treatment?

Partly

Is the background of the case’s history and progression described in sufficient detail?

Partly

Reviewer Expertise:

TB and MDR-TB

I confirm that I have read this submission and believe that I have an appropriate level of expertise to confirm that it is of an acceptable scientific standard, however I have significant reservations, as outlined above.

# Response

**Published:** 2025-10-06

**Authors:** Ajit Rayamajhi

**Affiliations:** Pediatrics, Kanti Children's Hospital, Kathmandu, Bagmati, Nepal

Thanks for sharing this detailed set of reviewer comments and your thoughtful responses. Here's a polished version of your replies with improved clarity, flow, and academic tone, while preserving your original intent: 1. Importance of History Taking and Early Suspicion of MDR-TB **Response:** Thank you for the comment. The case study underscores the critical role of thorough history taking, meticulous contact tracing, appropriate prophylaxis for MDR-TB contacts, and the need for wider availability of GeneXpert testing in resource-limited settings to facilitate timely diagnosis and treatment of TBM and other forms of TB in children. 2. Placement of Global Burden Data in the Introduction **Response:** Thank you for the suggestion. In the introduction, we have emphasized that MDR-TB—resulting from resistance to key first-line drugs—poses a significant challenge to TB control efforts in developing countries due to limited diagnostics, medication shortages, and mismanagement. Although rare in children, MDR-TB can lead to severe complications such as hydrocephalus and optic atrophy, reinforcing the need for early suspicion and prompt intervention. While the global burden data was initially placed in the discussion section, we have now expanded it—based on the first reviewer’s recommendation—to better integrate it into the discussion. For example: “Tuberculosis continues to be a major global health problem, affecting an estimated 10 million people annually, including 1.3 million children (WHO, 2023). Nearly two-thirds of new cases are concentrated in eight countries, most of which are in South Asia. Nepal reports 350–450 MDR-TB cases annually and was classified by WHO in 2023 among the top 30 countries with the highest MDR/RR-TB burden. While treatment success for drug-susceptible TB remains high, only 71% of MDR/RR-TB cases achieve successful outcomes.” 3. Terminology: “Major Anti-TB Drugs” vs. “Most Potent First-Line Anti-TB Drugs” **Response:** Thank you for the suggestion. We have revised the first sentence of the second paragraph in the introduction to: “If *Mycobacterium tuberculosis* becomes resistant to the most potent first-line antitubercular drugs such as rifampin and isoniazid, it can cause a disease known as multidrug-resistant tuberculosis (MDR-TB).” 4. Toxicity and Side Effects of MDR-TB Treatment **Response:** Thank you for the suggestion. We have incorporated this point into the first line of the third paragraph of the introduction: “In children, MDR-TB is rare and requires specialized clinical expertise for early detection and effective management. Moreover, the drugs used for treatment are often toxic, with long-term and potentially permanent side effects such as hearing loss.” 5. INH Prophylaxis Ineffectiveness and Recommended Regimen for MDR-TB Contacts **Response:** Thank you for the suggestion. We have added the following to the second paragraph of the discussion section on Page 6: “This case also highlights the ineffectiveness of isoniazid prophylaxis against MDR-TB. For contacts of MDR-TB cases, the recommended regimen includes 6 months of daily levofloxacin and 4 months of daily rifampicin.” 6. Updated WHO Estimate for Pediatric TB Burden **Response:** Thank you for the suggestion. We have updated the figure in the first line of the discussion section on Page 6 to reflect the latest WHO data: “Tuberculosis continues to be a major global health problem, affecting an estimated 10 million people annually, including 1.3 million children (https://www.who.int/news-room/fact-sheets/detail/tuberculosis).” 7. GeneXpert Availability and Systemic Barriers **Response:** Thank you for the suggestion. We have expanded the second paragraph of the discussion section to include the following: “Diagnosis of MDR-TB in children remains a significant challenge. In this case, earlier recognition of MDR-TB meningitis might have been possible with a more detailed exposure history and initial CSF GeneXpert testing. However, the test was temporarily unavailable due to a shortage of cartridges in the hospital supply chain, which was resolved only after logistical improvements. Delays in machine repair and lack of trained personnel also contribute to test unavailability. The father's history of drug-susceptible TB and prior isoniazid preventive therapy created a false sense of protection, delaying suspicion of resistant disease. Subsequent exposure history involving an uncle with MDR-TB clarified the diagnosis, underscoring the importance of detailed history taking and contact tracing. This case also reinforces the ineffectiveness of isoniazid prophylaxis against MDR-TB; instead, 6 months of daily levofloxacin and 4 months of daily rifampicin is recommended. Ultimately, limited access to GeneXpert testing and trained staff in resource-constrained settings continues to hinder timely diagnosis and treatment.”  
